# Biocompatible Fluorescent Core-Shell Nanoconjugates Based on Chitosan/Bi_2_S_3_ Quantum Dots

**DOI:** 10.1186/s11671-016-1417-6

**Published:** 2016-04-12

**Authors:** Fábio P. Ramanery, Alexandra A. P. Mansur, Herman S. Mansur, Sandhra M. Carvalho, Matheus C. Fonseca

**Affiliations:** Center of Nanoscience, Nanotechnology and Innovation - CeNano²I, Department of Metallurgical and Materials Engineering, Federal University of Minas Gerais, Av. Antônio Carlos, 6627-Escola de Engenharia, Bloco 2-Sala 2233, Belo Horizonte, Minas Gerais 31.270-901 Brazil; Department of Preventive Veterinary Medicine, UFMG, Belo Horizonte, Brazil; Department of Physiology and Biophysics, UFMG, Belo Horizonte, Brazil

**Keywords:** Nanoparticles, Nanoconjugates, Chitosan, Nanomaterial, Core-shell nanostructure

## Abstract

Bismuth sulfide (Bi_2_S_3_) is a narrow-bandgap semiconductor that is an interesting candidate for fluorescent biomarkers, thermoelectrics, photocatalysts, and photovoltaics. This study reports the synthesis and characterization of novel Bi_2_S_3_ quantum dots (QDs) functionalized using chitosan (CHI) as the capping ligands via aqueous “green” route at room temperature and ambient pressure. Transmission electron microscopy (TEM), UV-visible (UV-vis) spectroscopy, photoluminescence (PL) spectroscopy, dynamic light scattering (DLS), and zeta potential (ZP) analysis were used to characterize the hybrids made of biopolymer-functionalized Bi_2_S_3_ semiconductor nanocrystals. The results demonstrated that the CHI ligand was effective at nucleating and controlling the growth of water-soluble colloidal Bi_2_S_3_ nanoparticles. The average sizes of the Bi_2_S_3_ nanoparticles were significantly affected by the molar ratio of the precursors but less dependent on the pH of the aqueous media, leading to the formation of nanocrystals with average diameters varying from 4.2 to 6.7 nm. These surface-modified Bi_2_S_3_ nanocrystals with CHI exhibited photoluminescence in the visible spectral region. Moreover, the results of in vitro MTT (3-(4,5-dimethylthiazol-2yl)-2,5-diphenyltetrazolium bromide) assay with human osteosarcoma cells (SAOS) cell line demonstrated no cytotoxic response of the nanoconjugates.

Furthermore, the results indicated that the Bi_2_S_3_ QD–CHI nanoconjugates showed HEK293T cell uptake; therefore, they can be potentially used as novel fluorescent nanoprobes for the in vitro bioimaging of cells in biomedical applications.

**Graphical Abstract:**
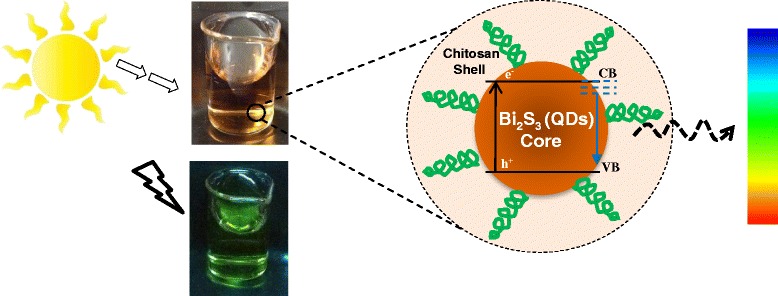
Schematic representation of the biocompatible core-shell nanostructure of the chitosan/Bi_2_S_3_ quantum dot conjugates with photoluminescent properties

Schematic representation of the biocompatible core-shell nanostructure of the chitosan/Bi_2_S_3_ quantum dot conjugates with photoluminescent properties

## Background

In recent years, the field of colloidal semiconductor nanocrystals, also referred to as colloidal quantum dots (QDs), has grown rapidly. The developments are the result of significant advances in nanoscience and nanotechnology that predominantly focus on biomedical and environmental applications [1]. The interdisciplinary contributions from several areas such as materials science, chemistry, and physics, combined with biology, pharmaceutics, medicine, and environmental science have created a fascinating new class of hybrid nanomaterials or nanoconjugates. These nanomaterials can be designed and engineered with almost any property or to carry out almost any function [2]. Basically, these nanosized conjugates combine the intrinsic functions of inorganic semiconductor nanomaterials and the versatile organic biointerfaces offered by polymers (e.g., chitosan, PVA, PEG) and biomolecules (e.g., amino acids, peptides, proteins, DNA) [3, 4]. In the realm of inorganic *low-dimensional* materials for producing nanoconjugates and nanostructures, QDs have been the major choice because of their unique combination of optical, electronic, magnetic, and chemical properties, which can be tuned via the modification of the nanoparticle size below the threshold value, named the Bohr radius [1, 5, 6]. In particular, the interest in narrow-bandgap materials such as bismuth chalcogenides (e.g., Bi_2_X_3_, X = S, Se, Te) nanocrystals has intensified in recent years [7, 8]. Studies from around the world suggest that these materials are realistic prospects for applications including solar cells, infrared optoelectronics (e.g., lasers, optical modulators, photodetectors, and photoimaging devices), low-cost/large-format microelectronics, and biological imaging and biosensor systems [7, 9].

However, due to their extremely low dimensions at the nanoscale and exceptionally high surface-area-to-volume ratio, these fluorescent nanocrystals must be stabilized by capping agents during their synthesis to restrict the growth of formed nuclei [10]. Hence, QDs have been produced using numerous processes such as in the pioneer studies entrapped in glasses [11, 12] or molecular films [13], encapsulated in polymer nanoparticles [14], dispersion in organic solvents [15], and colloidal dispersions [16, 17].

Nevertheless, despite almost three decades of advances in QD synthesis, the majority of the reported methods rely on organometallic processing routes that employ toxic solvents at high temperature and that lead to the formation of nanocrystals with hydrophobic surfaces [1, 5, 18]. Thus, synthesis of semiconductor QDs using an aqueous colloidal process is an attractive alternative to organometallic routes, which have received increasing concern because of their use of chemical processes that can be harmful to humans and to the environment [17, 19, 20]. In support of this, new environmentally friendly processes for producing QDs have been reported recently in the literature. Most studies employ the use of water-based colloidal routes using environmentally friendly and biocompatible reagents and precursors at low temperatures [17, 21]. In addition, colloidal chemistry provides a flexible platform for the surface functionalization of the QDs by applying an appropriate capping ligand, which in turn can simultaneously stabilize the inorganic semiconductor core of the nanoparticles and form an organic shell with biochemical functionalities for further applications [19].

Among several alternatives for capping agents, biopolymers, such as chitosan and its derivatives, have recently been proposed as a greener nanoplatform for producing water-soluble quantum dots. These polymers are intrinsically biocompatible, and they can be directly used as ligands for stabilizing QDs in aqueous media [22]. Chitosan is a natural biopolymer that is commonly produced from the alkaline deacetylation of chitin, which is mostly extracted from the exoskeleton of marine crustaceans. As a biopolymer, it has been broadly used in numerous biomedical and environmental applications due to its biocompatibility, biodegradability, commercial availability, and worldwide abundance associated with its eco-friendly properties [20]. Surprisingly, few studies have reported the preparation of nanomaterials based on bismuth sulfide (Bi_2_S_3_), such as quantum dots [7] and nanorods [9, 23], but no research investigating Bi_2_S_3_–chitosan nanoconjugates was found in the consulted literature.

Thus, in this study, new carbohydrate-based nanoconjugates combining chitosan with Bi_2_S_3_ semiconductor QDs were designed and synthesized via a single-step “green” aqueous colloidal process at room temperature. The results demonstrated that chitosan was an effective polymer ligand for nucleating and stabilizing ultra-small Bi_2_S_3_ QDs, forming colloidal core-shell nanostructures in aqueous dispersions. In addition, it was verified that variation of pH and molar ratio of precursors during the synthesis affected the physico-chemical properties and morphological aspects of the nanostructures. Moreover, these water-soluble nanoconjugates were photoluminescent under light irradiation and biocompatible toward SAOS cell culture, which can be potentially used as narrow-bandgap fluorophores in biomedical and pharmaceutical applications using an environmentally friendly process.

## Methods

### Materials

All of the reagents and precursors, including bismuth chloride (Aldrich, USA, ≥ 98%, BiCl_3_), sodium sulfide (Synth, Brazil, > 98 %, Na_2_S·9H_2_O), sodium hydroxide (Merck, USA, ≥ 99 %, NaOH), and acetic acid (Synth, Brazil, ≥ 99.7 %, CH_3_COOH) were used as received. Chitosan (Aldrich Chemical, USA, catalogue # 419419; high molecular weight, *M*_W_ = 310 to > 395 kDa; degree of deacetylation DD ≥ 75.0 %; viscosity 800–2000 cP, 1 wt% in 1 % acetic acid) was used as the reference polysaccharide ligand. Unless otherwise indicated, deionized water (DI water, Millipore Simplicity™) with a resistivity of 18 MΩ cm was used to prepare the solutions, and the procedures were conducted at room temperature (RT, 23 ± 2 °C).

### Methods

#### Synthesis of Bi_2_S_3_/Chitosan Nanoconjugates

Bi_2_S_3_ nanoparticles were synthesized via an aqueous colloidal route in a reaction flask at room temperature. Precursors with three different molar ratios, [Bi^3+^]/[S^2−^], were evaluated: 0.33 (excess of sulfur), 0.67 (stoichiometric), and 1.33 (excess of bismuth). The synthesis of the Bi_2_S_3_ nanoparticles was carried out as follows: 2 mL of chitosan (CHI) solution (1 % w/v in 2 % v/v aqueous solution of acetic acid) and 45 mL of DI water were added to the flask reaction vessel. Under moderate magnetic stirring, *X* mL (*X* = 6 mL for 0.33; *X* = 3 mL for 0.67 and 1.33) of S^2−^ precursor solution (Na_2_S·9H_2_O, 1.0 × 10^−2^ mol L^−1^) and *Y* mL (*Y* = 2 mL for 0.33 and 0.67; *Y* = 4 mL for 1.33) of Bi^3+^ precursor solution (BiCl_3_, 1.0 × 10^−2^ mol L^−1^ in acetic acid) were added to the flask and stirred for 60 min. During the addition of Bi^3+^ solution, the pH was measured and adjusted to 2.5 ± 0.1 or 3.5 ± 0.1 with NaOH (1.0 mol L^−1^). The Bi_2_S_3_ QDs suspensions produced were referred to as QD_CHI[Bi^3+^]/[S^2−^]_pH, where [Bi^3+^]/[S^2−^] was 0.33, 0.67, or 1.33 and the pH was 2.5 or 3.5, as a function of the molar ratio of precursors and the pH of quantum dots synthesis.

#### Characterization of Bi_2_S_3_/Chitosan Nanoconjugates

UV-visible (UV-vis) spectroscopy measurements were conducted using Perkin-Elmer equipment (Lambda EZ-210) in transmission mode with a quartz cuvette. Measurements were taken over a wavelength range of 1100 to 190 nm.

The morphological and structural features of the quantum dots were characterized using transmission electron microscopy (TEM, Tecnai G2-20-FEI microscope, 200 kV) coupled to an energy dispersive X-ray (EDX) microprobe and using selected area electron diffraction (SAED) analysis. QD sizes and distribution data were obtained based on the TEM images by measuring at least 100 randomly selected nanoparticles using an image processing freeware program (ImageJ, version 1.49, public domain, National Institutes of Health).

X-ray photoelectron spectra (XPS) analysis was performed on an Amicus spectrometer (Shimadzu, Japan) using Mg-Kα as the excitation source. All peaks positions were corrected based on C 1s binding energy (284.6 eV).

Dynamic light scattering (DLS) and zeta potential (ZP) measurements were performed in the QD colloidal dispersions using a ZetaPlus instrument with the laser light diffusion method (Brookhaven Instruments).

Photoluminescence (PL) characterization of the nanohybrids was conducted based on spectra acquired at room temperature using a violet diode laser module at *λ*_exc_ = 405 nm (150 mW, Roithner LaserTechnik, GmbH) coupled to a USB4000 VIS-NIR spectrophotometer (Ocean Optics).

In addition, the QD colloidal solutions were placed inside a “darkroom-chamber” where they were illuminated by a UV radiation emission bulb (*λ*_excitation_ = 365 nm, 6-W, Boitton Instruments). Digital color images were collected of the fluorescence of the QDs in the visible range of the light spectrum. Quantum yield (QY) was measured according to established procedure by using Rhodamine 6G (Sigma, USA) in ethanol as the standard at *λ*_excitation_ = 405 nm [24].

#### Cytotoxicity Assay by MTT

##### Culture of Human Sarcoma Cell Line Culture (SAOS)

The immortalized human osteosarcoma-derived (SAOS) cells were provided by Prof. A. Goes of the Department of Immunology and Biochemistry, Universidade Federal de Minas Gerais (UFMG). SAOS cells are broadly accepted as a model cell line for the preliminary assessment of biocompatibility of materials and devices. The SAOS cells were cultured in DMEM (Dulbecco’s modified eagle medium) with 10 % fetal bovine serum (FBS), streptomycin sulfate (10 mg mL^−1^), penicillin G sodium (10 units mL^−1^), and amphotericin-b (0.025 mg mL^−1^), all of them were supplied by Gibco BRL (NY, USA), using a humidified atmosphere of 5 % CO_2_ at 37 °C. The cells were used for experiments on passage 23.

All of the biological tests were performed according to ISO standards 10993-5:1999 (Biological evaluation of medical devices; part 5: tests for in vitro cytotoxicity)*.* All experiments were performed using the direct contact methodology.

##### Method

SAOS cells were plated (3 × 10^5^ cells/well) in 96-well plates. Cell populations were synchronized in serum-free media for 24 h. After this period, the media volume was suctioned and replaced with media containing 10 % FBS for 24 h. The samples of Bi_2_S_3_/polymer nanoconjugates were added to individual wells at a concentration of 3.0 %. Controls had been used with cells and DMEM medium with 10 % FBS, positive control Triton X-100 (1 %) from Sigma-Aldrich (St. Louis, MO, USA) and as negative control chips sterile polypropylene Eppendorf (1 mg mL^−1^, Eppendorf, Hamburg, Germany). After 24 h, all media was aspirated and replaced with 60 μL culture medium with serum to each well and photographed using an inverted optical microscope (Leica DMIL LED, Germany). MTT (3-(4,5-dimethylthiazol-2yl)-2,5-diphenyltetrazolium bromide) (5 mg mL^−1^, Sigma-Aldrich, St. Louis, MO, USA) were added to each well and incubated for 4 h in an oven at 37 °C and 5 % CO_2_. After, they were placed on a 40-μL SDS solution/4 % HCl, with incubation for 16 h in an oven at 37 °C and 5 % CO_2_. Then, 100 μL were removed from each well and transferred to a 96-well plane, and quantifying of the absorbance was taken into a Thermo Plate (TP-READER) with a 595-nm filter. The values obtained were expressed as percentage of viable cells according to Eq. 1. It is attributed that the values of controls (wells with cells, and no samples) has 100 % cell viability.

1 $$ \mathrm{Cell}\ \mathrm{viability} = \frac{\mathrm{Abs}\ \mathrm{of}\ \mathrm{sample}\ \mathrm{and}\ \mathrm{cells}}{\mathrm{Abs}\ \mathrm{of}\ \mathrm{control}} \times 100\% $$

Prism software (GraphPad Software, San Diego, CA, USA) was used for data analysis. Statistical significance was tested using one-way ANOVA followed by Bonferroni method, with *p* < 0.05 considered statistically significant. The experiments were performed in triplicate (*n* = 3).

#### Cellular Uptake of Bi_2_S_3_ Quantum Dot/Chitosan Nanoconjugates

##### Kidney Cell Line of a Human Embryo Culture (HEK293T Cells)

The human embryonic kidney cell line (HEK293T) was kindly provided by Prof. M.F. Leite of the Department of Physiology and Biophysics, UFMG. The cells were cultured in DMEM with 10 % FBS, penicillin G sodium (10 units mL^−1^), streptomycin sulfate (10 mg mL^−1^), and amphotericin-b (0.025 mg mL^−1^), in a humidified atmosphere of 5 % CO_2_ at 37 °C. The HEK293T cells were used for the experiments on passage 7.

##### Confocal Laser Scanning Microscopy

The HEK293T cells were plated (5 × 10^4^ cells/well) in 24-well plates. The cells were incubated for 4 days in 5 % CO_2_ at 37 °C and synchronized for 24 h. The QD_CHI0.67_2.5 sample containing 50 % of the medium solution was added to the HEK293T cells. Next, the cells were incubated in 5 % CO_2_ at 37 °C for 1 h and washed with phosphate-buffered saline (PBS, Gibco BRL, NY, USA). After washing, the cells were fixed with paraformaldehyde (4 %) for 30 min and washed three times with PBS, and cover slips were mounted with Hydromount (Fisher Scientific Ltd., Leicestershire, UK). Confocal laser scanning fluorescence microscopy (Zeiss LSM Meta 510, Carl Zeiss, Germany) was used to detect the fluorescence of the cells using a 488-nm argon laser irradiation to excite the QD–chitosan nanoconjugates. The emissions were collected in the range between 505 and 530 nm. For the control, HEK293T cells were incubated with only DMEM medium with 10 % FBS (immunofluorescence).

## Results and Discussion

### Physico-chemical Characterization of Chitosan/Bi_2_S_3_ Nanoconjugates

**Fig. 1:**
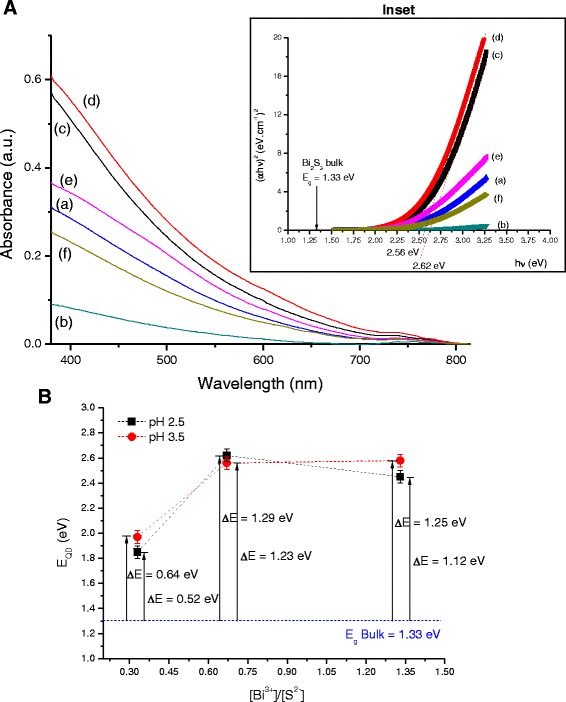
**a** UV-vis spectra of Bi_2_S_3_ systems: (*a*) QD_CHI0.33_2.5, (*b*) QD_CHI0.33_3.5, (*c*) QD_CHI0.67_2.5, (*d*) QD_CHI0.67_3.5, (*e*) QD_CHI1.33_2.5, and (*f*) QD_CHI1.33_3.5. *Inset*: optical bandgap using “TAUC” relation. **b** Blue-shift value as a function of pH (*black squares*, pH 2.5, and *red circles*, pH 3.5) and molar ratio of precursors ([Bi^3+^]/[S^2−^]). A two-column figure

Figure 1a shows the UV-vis absorption curves of the Bi_2_S_3_ nanoparticles stabilized using chitosan as the capping ligand. An absorbance onset was observed at approximately *λ*=800 nm. In general, the UV-vis spectra did not present clearly defined excitonic peaks (i.e., broad excitonic transitions), which may be associated with the relative dispersity of QD size characteristic of aqueous colloidal processing routes compared to organometallic methods [1]. The bandgap energy (*E*_QD_) of the synthesized nanoparticles was estimated using the linear form of TAUC relation for direct bandgap semiconductors (Eq. 2) [25] that relates the absorption coefficient (α) and the photon energy (hν) using a band form parameter (B).

2 $$ {\left(\mathsf{\alpha}\mathsf{h}\mathsf{\nu}\right)}^{\mathsf{2}}=\mathsf{B}\left(\mathsf{h}\mathsf{\nu}\hbox{-} {\mathsf{E}}_{\mathsf{QD}}\right) $$

The direct bandgap value of Bi_2_Si_3_ QDs was calculated from the plots of (αhν)^2^ versus hν extrapolating the straight portion of the graph (inset of Fig. 1a) to αhν = 0. The obtained *E*_QD_ values (Fig. 1b) were higher than the corresponding bulk value (1.33 eV) for bismuth sulfite [26] as a consequence of the size quantization characteristics of the nanoparticles produced. The “blue-shift” (Δ*E* = *E*_QD_ − *E*_g_) determined for the QDs compared to the bulk value is presented in Fig. 1b.

**Fig. 2:**
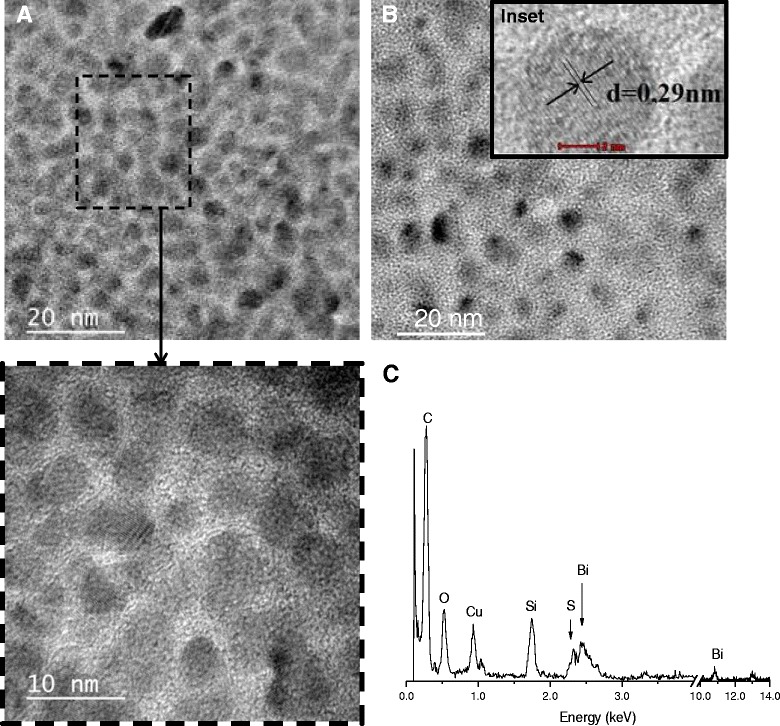
TEM images of **a** QD_CHI0.67_2.5 and **b** QD_CHI0.67_3.5 (*inset*: SAED pattern). **c** Typical EDX spectrum. A two-column figure

**Fig. 3:**
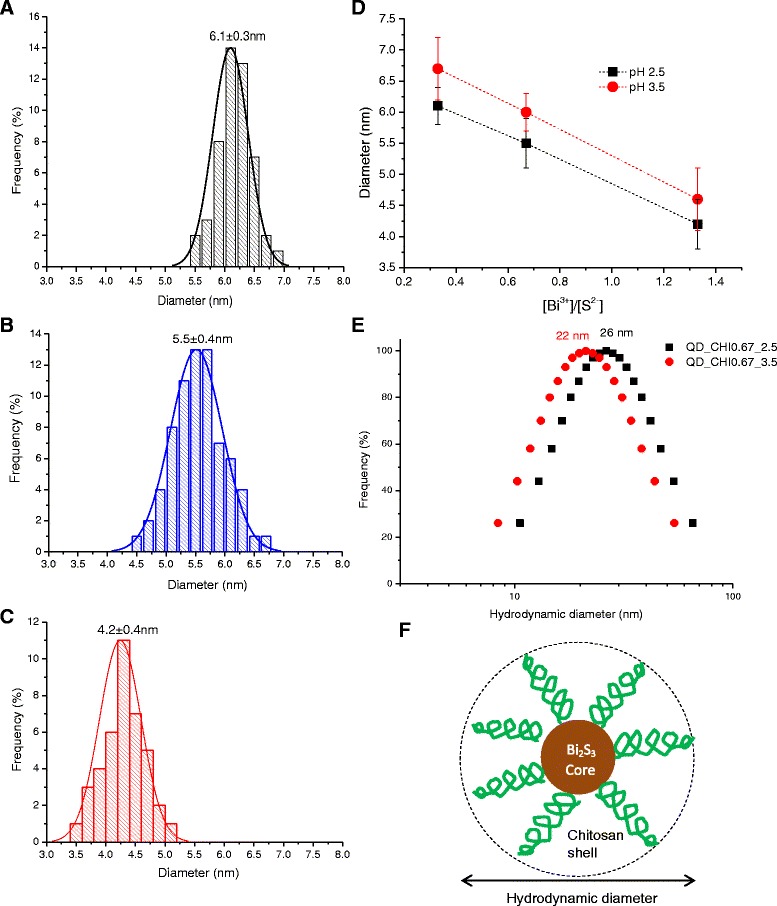
Histograms of size distribution of **a** QD_CHI0.33_2.5, **b** QD_CHI0.67_2.5, and **c** QD_CHI1.33_2.5. **d** Summary of QD diameter as a function of pH (*black squares*, pH 2.5, and *red circles*, pH 3.5) and molar ratio of precursors ([Bi^3+^]/[S^2−^]). **e** DLS results for QD_CHI0.67_2.5 (*black squares*) and QD_CHI0.67_3.5 (*red circles*). **f** Schematic representation of core-shell structure of the nanoconjugate (not to scale). A two-column figure

Typical TEM image obtained for the synthesized samples are shown in Fig. 2a, b, revealing that Bi_2_S_3_ QDs were spherical and homogenously dispersed. It can be noted that the nanoparticle sizes were below Bohr’s radius (*a*_B_ ~24 nm) for Bi_2_S_3_ [26], confirming that nanoparticles were in the “quantum confinement regime” and properly stabilized by chitosan as the polymer capping ligand. SAED patterns (inset of Fig. 2b) indicated a lattice parameter of 0.29 ± 0.1 nm compatible with the (221) plane of bismuthinite (JCPDS-43-1471). The EDX spectrum clearly indicated Bi and S chemical elements in addition to the peaks related to chitosan, the grid, and the detector (i.e., C, Si, O). The average Bi_2_S_3_ nanoparticle sizes prepared at pH 2.5 were evaluated using TEM images. Histograms of the diameter distribution for the samples with three molar ratios of [Bi^3+^]/[S^2−^] = 0.33, 0.67, and 1.33 are shown in Fig. 3a–c, respectively. Based on the results, the molar ratio of the precursors considerably influenced the nanoparticle size of QDs with the highest content of bismuth ([Bi^3+^]/[S^2−^] = 1.33, i.e., excess of Bi) leading to the formation of the smallest nanocrystal size. The same trend was observed for the syntheses performed at pH 3.5 (Fig. 3d). Therefore, differences in the pH did not considerably alter the Bi_2_S_3_ QD size. However, it is important to point out that the size of Bi_2_S_3_ nanoparticles for higher molar ratios (i.e., [Bi^3+^]/[S^2−^] > 1.33), which were not synthesized in this study cannot be directly predicted or extrapolated based on the results displayed in Fig. 3d of the three molar ratios of [Bi^3+^]/[S^2−^] (0.33, 0.67, 1.33). Essentially, there are several complex thermodynamics and kinetics aspects involved in the colloidal system, regarding the nucleation/growth of these ultra-small nanocrystals in aqueous media using chitosan as a pH-dependent and multidentate polymeric ligand. It can be expected that the dimension of the Bi_2_S_3_ nanocrystals will not reduce much further at higher [Bi^3+^]/[S^2−^] ratios, considering the relative depletion of [S^2−^] caused by the excess of [Bi^3+^] in the medium, decreasing the kinetics of the reaction. In addition, an exponential increase on the surface-area-to-volume ratio occurs by reducing the size of the nanoparticles therefore, disfavoring the thermodynamics for nucleation (+∆*G*).

The effect of the molar ratio ([Bi^3+^]/[S^2−^]) on nanoparticle size may be explained as follows: at pH = 2.5 and 3.5, all of the amine groups of chitosan are protonated [27] and negatively charged sulfides (S^2−^) interact with –NH_3_^+^ groups as represented in Eqs. 3–5. When Bi^3+^ ions were added to the reacting vessel, they acted as nucleation sites for Bi_2_S_3_ particle formations (Eq. 6). The excess of bismuth at a molar ratio of [Bi^3+^]/[S^2−^] = 1.33 favored the stabilization of the Bi_2_S_3_ nanocrystals at smaller dimensions due to the increase in the number of nucleation sites (“seeds”). In parallel, the lower ratio of [Bi^3+^]/[S^2−^] produced relatively larger Bi_2_S_3_ QDs, as the S^2−^ species in solution were stabilized by the protonated amine groups in the chitosan chain reducing the nucleation kinetics.

3 $$ \mathrm{CHI}\hbox{-} {\mathrm{NH}}_{2\left(\mathrm{a}\mathrm{q}\right)} + {{\mathrm{H}}^{+}}_{\left(\mathrm{a}\mathrm{q}\right)}\to\ \mathrm{CHI}\hbox{-} {\mathrm{NH}}_3{{}^{+}}_{\left(\mathrm{a}\mathrm{q}\right)} $$

4 $$ {\mathrm{Na}}_2{\mathrm{S}}_{\left(\mathrm{a}\mathrm{q}\right)}\to\ {{2\mathrm{N}\mathrm{a}}^{+}}_{\left(\mathrm{a}\mathrm{q}\right)} + {{\mathrm{S}}^{2-}}_{\left(\mathrm{a}\mathrm{q}\right)} $$

5 $$ 2\mathrm{CHI}\hbox{-} {\mathrm{NH}}_3{{}^{+}}_{\left(\mathrm{a}\mathrm{q}\right)} + {{\mathrm{S}}^{2-}}_{\left(\mathrm{a}\mathrm{q}\right)}\ \to\ 2\left({{\mathrm{CHI}\hbox{-} \mathrm{N}\mathrm{H}}_3}^{+}\right) - - - {\left({\mathrm{S}}^{2-}\right)}_{\left(\mathrm{a}\mathrm{q}\right)} $$

6 $$ 2\ \left({{\mathrm{CHI}\hbox{-} \mathrm{N}\mathrm{H}}_3}^{+}\right) - - - {\left({\mathrm{S}}^{2-}\right)}_{\left(\mathrm{a}\mathrm{q}\right)} + {\mathrm{Bi}}^{3+}\to\ {\mathrm{Bi}}_2{\mathrm{S}}_{3\left(\mathrm{s}\right)}\kern1.5em  + {{\mathrm{CHI}\hbox{-} \mathrm{N}\mathrm{H}}_3}^{+} $$

DLS results (Fig. 3e) revealed that the hydrodynamic diameter (*H*_D_) of the core-shell (Bi_2_S_3_–chitosan) nanoconjugates were approximately 22 and 26 nm for the systems QD_CHI0.67_2.5 and QD_CHI0.67_3.5, respectively. These values are associated with the interactions between chitosan and aqueous medium (Fig. 3f). As expected, the *H*_D_ values are higher than the sizes evaluated by TEM technique, which were only related to dimension of the inorganic core of Bi_2_S_3_ nanocrystals.

**Fig. 4:**
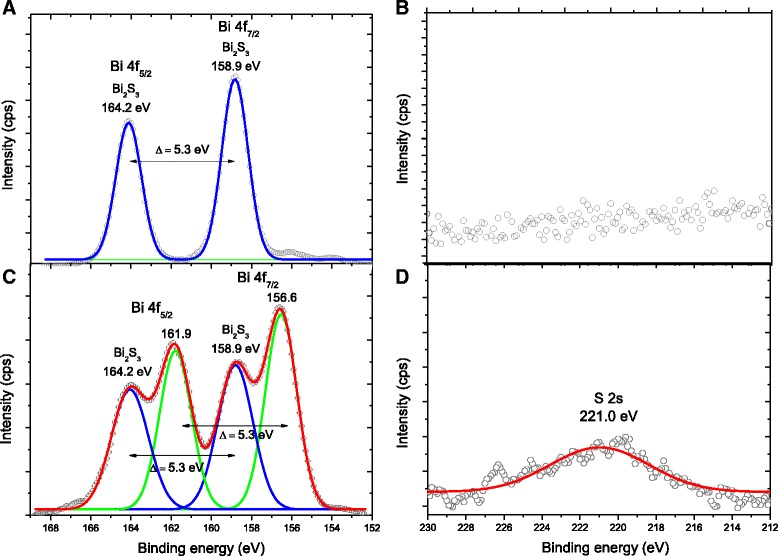
XPS spectra of Bi_2_S_3_ nanoconjugate (QD_CHI0.67_3.5): Bi 4f region (**a**) before and (**c**) after Ar^+^ etching and S 2s region (**b**) before and (**d**) after Ar^+^ etching. A two-column figure

To perform a more in-depth characterization of the Bi_2_S_3_ QDs at the nanoscale order and to identify the chemical states of the elements at the surfaces, the nanoconjugates were evaluated by XPS. Figure 4a shows typical XPS spectra obtained directly at the surface of the Bi_2_S_3_/chitosan nanoconjugate. The peaks at 158.9 and 164.2 eV correspond to the Bi 4f_7/2_ and Bi 4f_5/2_ levels, respectively, which are generally assigned to Bi–S bonding in Bi_2_S_3_ [28, 29]. After etching (Fig. 4c, Ar^+^, 1 cycle, 60 s, emission current 10 mA, and beam voltage 0.5 kV), the Bi 4f_7/2_ consisted of two binding energies of 156.6 and 158.9 eV, whereas the binding energies values for Bi 4f_5/2_ were centered at 161.9 and 164.2 eV. The peaks at 156.6 and 161.9 eV were assigned to bismuth under-coordinated and reduced species due to the etching by argon ion beam [28, 29]. The peaks at 158.9 and 164.2 eV indicate the presence of Bi^3+^ in the Bi_2_S_3_ phase, as already observed at the surface prior to etching. In addition, the XPS spectra at the QD surface in the S 2s region (Fig. 4b) indicated no detectable signal from sulfur. However, after etching, the surface showed a broad peak approximately at 221.0 eV, which can be assigned to the binding energy of S^2−^ state in Bi_2_S_3_ [30]. This result indicated that Bi-rich surfaces of Bi_2_S_3_ quantum dots were produced, which is consistent with metal-chalcogenide nanocrystals synthesized in the presence of coordinating ligands bound covalently to cations [7].

Zeta potential (ζ) measurements for QD_CHI0.67_2.5 and QD_CHI0.67_3.5 were ζ = +56 ± 1 mV and ζ = +37 ± 3 mV, respectively. Under the highly acidic conditions of both pH values investigated, all amine groups of chitosan are protonated, but at pH 2.5, there are more H^+^ ions in solution repelling and exposing more –NH_3_^+^ groups at the nanohybrid surface (i.e., the outermost surface of the organic shell). At pH 3.5, the molar ratio of the precursors also influenced the ZP values. At a molar ratio [Bi^3+^]/[S^2−^] = 0.33, ZP was measured to be ζ = +31 ± 3 mV, which is lower than the value obtained for the stoichiometric system where [Bi^3+^]/[S^2−^] = 0.67 (i.e., Bi_2_S_3_, ratio 2/3 = 0.67). This difference may be due to the excess S^2−^ ions forming complexes with protonated amines reducing the positive charge at the double layer. For the system QD_CHI1.33_3.5, ZP was ζ = +33 ± 11 mV. The relatively higher statistical standard deviation may be attributed to the excess of Bi^3+^ species that increases the repulsion between the –NH_3_^+^ groups in the chitosan chains, thus disrupting the balanced core-shell nanostructure. In addition, ZP results indicated that the QDs were mostly electrostatically stabilized (ζ > +30 mV), preventing close contact between nanoparticles, compatible with the homogenously dispersed features of nanoparticles observed in TEM images. As a general trend, irrespective to the molar ratio of Bi:S used in the synthesis, all nanoconjugates presented positive ζ-potential values, which was attributed to the protonation of amine groups of the chitosan polymeric shell under the acidic conditions at pH = 2.5 and pH = 3.5. Clearly, it is worth mentioning that, as far as the potential applications of these nanoconjugates in biomedical and environmental fields are concerned, these colloidal systems need to be previously buffered to raise the pH of the media to the range of 6.0 to 8.0, to render them biocompatible.

**Fig. 5:**
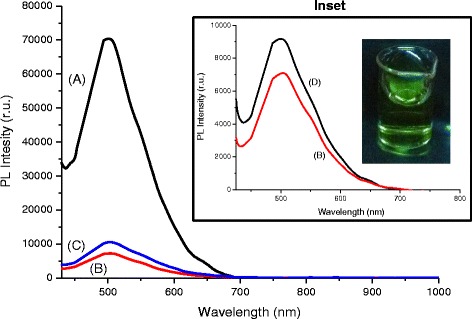
PL spectra of Bi_2_S_3_ nanoconjugates: (*a*) QD_CHI0.33_3.5, (*b*) QD_CHI0.67_3.5, (*c*) QD_CHI1.33_3.5, and (*d*) QD_CHI0.67_2.5 (*λ*_excitation_ = 405 nm). *Inset*: effect of pH in PL emission and green luminescence of Bi_2_S_3_ quantum dots under UV excitation (“dark chamber”). A two-column figure

All of the systems presented luminescent behavior under excitation, with the predominant PL emission band appearing in the spectral range of 450 to 650 nm (green-orange) (Fig. 5 and inset). The results of photoluminescence of Bi_2_S_3_ nanoconjugates indicated that the excitonic emission is not predominant, because the maxima wavelengths of the PL spectra were statistically similar (*λ*_PL_ = 504 ± 1 nm) despite the different absorptions in UV-vis spectroscopy. Thus, the emission is the overall contribution of radiative recombination from QDs and/or electronic transitions from Bi^3+^, usually the backward radiative transition ^3^P_1_→^1^S_0_ [31]. Based on Fig. 5, the luminescence intensity was reasonably dependent on the molar ratio of precursors: the highest luminescence response was measured for the 0.33 ratio, which is associated with the biggest nanocrystal size. This behavior is usually observed due to the density of surface defects (e.g., energy trap stages) and increase in the non-radiative pathways as the quantum dot size decreases [1, 32]. In addition, the QY of the Bi_2_S_3_ nanoconjugates was estimated to be approximately 1.0 %, in good agreement with the reports published of QDs synthesized using aqueous colloidal routes at low temperatures, which is usually smaller than of QDs prepared at high temperature in organic process [1].

### Cytotoxicity Assay by MTT of Chitosan/Bi_2_S_3_ Nanoconjugates

The biocompatibility of Bi_2_S_3_ nanomaterials has been reported in the literature [33–35]. In this study, the assessment of the cytotoxicity of core-shell Bi_2_S_3_/chitosan nanoconjugates was performed using the enzymatic-based MTT assay, with samples synthesized at pH = 3.5, (QD_CHI0.67_3.5) using SAOS cell line. This method is considered superior to other similar methods because it is safe, easy to use, has a high reproducibility, and is broadly performed for both cell viability and cytotoxicity tests.

**Fig. 6:**
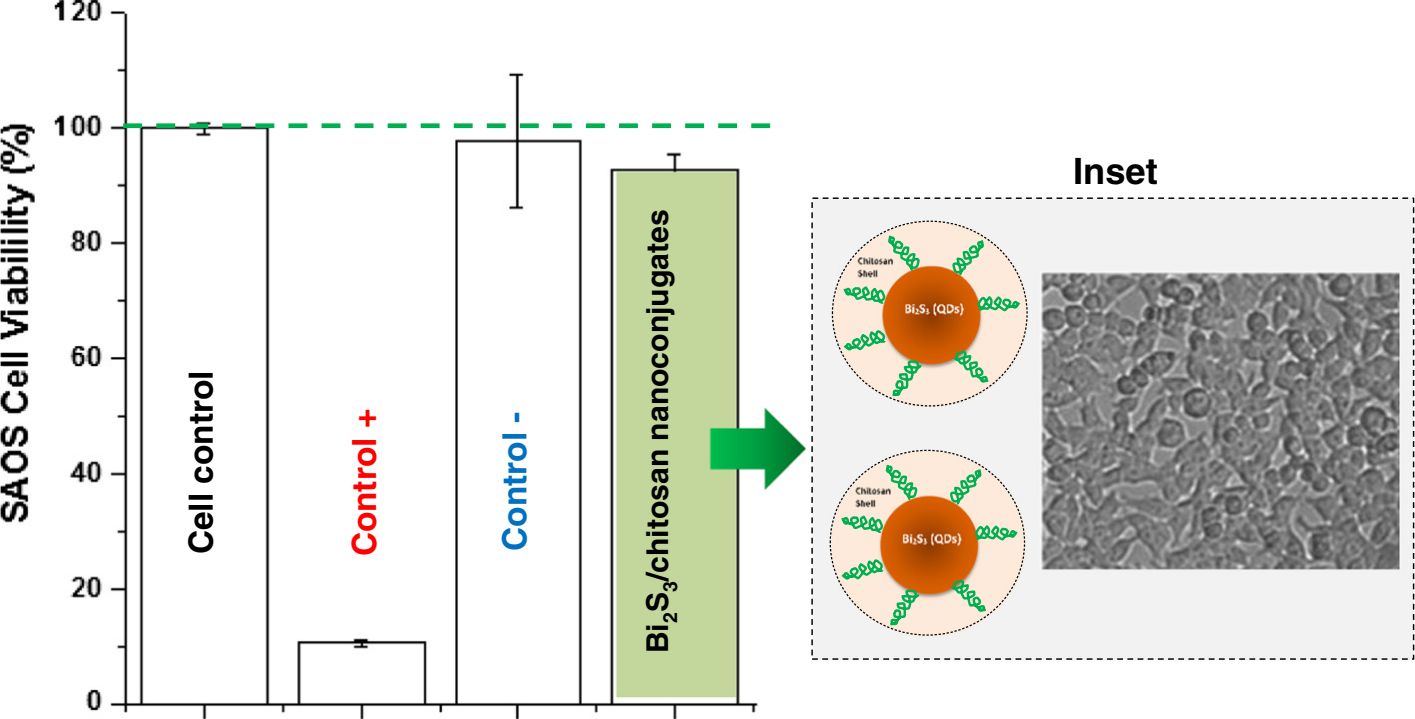
Cell viability response of SAOS culture using the MTT assay of chitosan/Bi_2_S_3_ nanoconjugate (QD_CHI0.67_3.5) after 24 h of incubation. *Inset*: schematic representation of the interactions of nanoconjugates at the cell biointerface (not to scale). A two-column figure

Thus, the results of SAOS cells in contact with the Bi_2_S_3_/chitosan nanoconjugate samples evidenced no difference in the cell viability and non-toxic effect compared to the control group, within the statistical range of variation (Fig. 6). The nanoconjugates showed cell viability response of over 90 % (i.e., non-cytotoxic), which can be essentially assigned to the approach used in this research. That means, these nanoconjugates were designed and produced with a “cadmium-free” nanocore (Bi_2_S_3_) and surface functionalized with the chitosan polysaccharide shell aiming at rendering them biocompatible and, therefore, theoretically safer for biological applications. The schematic representation of interactions of the nanoconjugates with the SAOS cell membranes is depicted in Fig. 6 (inset). Moreover, these novel biocompatible and water-soluble nanoconjugates were developed using a facile eco-friendly aqueous processing route.

### Cell Uptake of Bi_2_S_3_/Chitosan Nanoconjugates Biomarkers

**Fig. 7:**
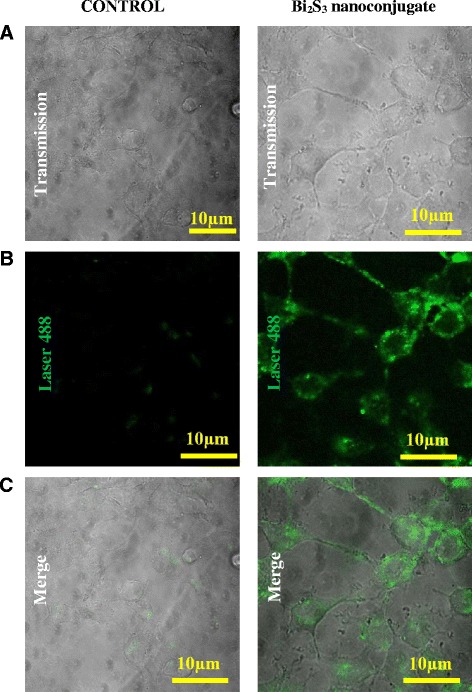
Confocal microscopy bioimaging of the cellular uptake of the Bi_2_S_3_/chitosan nanoconjugates by HEK293T cells in comparison to the control condition. **a** Bright field. **b** Fluorescence image. **c** Mapping of the distribution of nanoconjugates inside the cells (630×). A two-column figure

Endocytosis is the biological process responsible for the internalization of nanoparticles by cells. This process depends on energy and functional coordination of lipids and plasma membrane. Therefore, studies that allow the visualization of internalization of nanomaterials are important tools for the development and biological characterization of nanoparticles [36, 37]. Thus, in this study, confocal fluorescence microscopy was used to determine the cell uptake of the Bi_2_S_3_ nanoconjugates in HEK293T cells. Figure 7 shows fluorescent images of QD_CHI0.67_3.5 internalization in comparison with the control sample (autofluorescence). These results demonstrated the biocompatibility and bioimaging properties of the novel Bi_2_S_3_ QD–chitosan nanoconjugates. They can be used for intracellular targeting as biomarker or for drug-delivery because these nanoconjugates presented efficient translocation across the cell plasma membrane [38].

## Conclusions

In summary, nanoconjugates were designed and synthesized with chitosan as the biopolymer shell and Bi_2_S_3_ semiconductor quantum dot as the fluorescent inorganic core. These nanohybrids were produced using a single-step eco-friendly aqueous colloidal processing route at room temperature. The results demonstrated that chitosan behaved as an effective ligand for nucleating and stabilizing ultra-small Bi_2_S_3_ QDs, leading to the formation of colloidal core-shell nanostructures in aqueous dispersions. These nanoconjugates were cytocompatible evaluated by the MTT assay and exhibited photoluminescence under light excitation. Furthermore, the results of cell studies show that the QD–chitosan conjugates display good HEK293T cell uptake in the absence of non-specific binding to the cell membrane and, therefore, they can be potentially used as fluorescent nanosized bioprobes to label cells in vitro for cell bioimaging applications.
